# An *In Vitro* Model for Oral Mixed Biofilms of *Candida albicans* and *Streptococcus gordonii* in Synthetic Saliva

**DOI:** 10.3389/fmicb.2016.00686

**Published:** 2016-05-12

**Authors:** Daniel Montelongo-Jauregui, Anand Srinivasan, Anand K. Ramasubramanian, Jose L. Lopez-Ribot

**Affiliations:** ^1^Department of Biology, The University of Texas at San AntonioSan Antonio, TX, USA; ^2^South Texas Center for Emerging Infectious Diseases, The University of Texas at San AntonioSan Antonio, TX, USA; ^3^Department of Biomedical Engineering, The University of Texas at San AntonioSan Antonio, TX, USA

**Keywords:** *Candida albicans*, *Streptococcus gordonii*, mixed biofilms, synthetic saliva

## Abstract

As a member of the normal human oral microbiota, the fungus *Candida albicans* is often found in association with *Streptococcus gordonii*, a member of dental plaque forming bacteria. Evidence suggests that *S. gordonii* serves as a facilitator of *C. albicans* adherence to dental tissues, which represents a clinically relevant problem, particularly for immunocompromised individuals that could subsequently develop fungal infections. In this study we describe the development of a relatively simple and economical *in vitro* model that allows for the growth of mixed bacterial/fungal biofilms in 96-well microtiter plates. We have applied this method to test and compare the growth characteristics of single and dual species biofilms in traditional microbiological media versus a synthetic saliva medium (basal medium mucin, BMM) that more closely resembles physiological conditions within the oral cavity. Results indicated a synergistic effect for the formation of biofilms when both microorganisms were seeded together under all conditions tested. The structural and architectural features of the resulting biofilms were further characterized using scanning electron microscopy and confocal scanning laser microscopy. We also performed drug susceptibility assays against single and mixed species biofilms using commonly used antifungals and antibacterial antibiotics, both in monotherapy and in combination therapy, for a direct comparison of resistance against antimicrobial treatment. As expected, mixed species biofilms displayed higher levels of resistance to antimicrobial treatment at every dose tested in both traditional media and BMM synthetic saliva, as compared to single-species biofilms.

## Introduction

*Candida albicans* is an opportunistic pathogenic fungus able to colonize and cause infections in a variety of host sites, including the oral cavity. As such, candidiasis represents the most common oral fungal infection affecting mostly immunosuppressed patients, denture wearers and the elderly (Muzyka, 2005; Meurman and Hamalainen, 2006; Muzyka and Epifanio, 2013). Oral sites that *C. albicans* is able to colonize and subsequently cause infection include mucosal surfaces, periodontal pockets, root canals, enamel, dentures and ortho dontic appliances (Ramage et al., 2004; de Carvalho et al., 2006; Arslan et al., 2008; Dongari-Bagtzoglou et al., 2009; Sardi et al., 2010; Freitas et al., 2014). Most frequently oral candidiasis is associated with the formation of *C. albicans* biofilms, leading to high levels of resistance to antimicrobial therapy and providing protection against the host’s immune system, thus further complicating treatment (Hawser and Douglas, 1995; Williams et al., 2011; Mathe and Van Dijck, 2013). In recent years there has been an increased recognition of the complexity of these biofilms, which often are polymicrobial in nature as a result of the interactions between *C. albicans* and members of the oral bacterial microbiome (Hwang et al., 2015; Jakubovics, 2015; O’Donnell et al., 2015a,b; Wu et al., 2015). Streptococci of the mitis group, most notably *Streptococcus gordonii*, are among the early colonizers and comprise a large proportion of the oral microbiota, and the ability of *C. albicans* to interact with oral streptococci and form biofilm consortia in multiple oral sites has been documented (Jenkinson et al., 1990; Bamford et al., 2009; Silverman et al., 2010; Falsetta et al., 2014; Jakubovics et al., 2014; Xu et al., 2014; Chukkapalli et al., 2015). These interactions are bidirectional and considered to be mutualistic beneficial, leading to a cooperative relationship that greatly contributes to survival, persistence and pathogenicity of these microorganisms in diverse oral niches (Jenkinson et al., 1990; Waltimo et al., 1997; O’Sullivan et al., 2000; Lana et al., 2001; Jenkinson and Douglas, 2002; Holmes et al., 2006; Shirtliff et al., 2009; Morales and Hogan, 2010).

A variety of *in vitro* models have been developed and used to study and characterize mixed fungal/bacterial biofilms. Virtually all these models use traditional nutrient-rich microbiological media that have been optimized for either bacterial or fungal growth (Jenkinson et al., 1990; Holmes et al., 1995; Bamford et al., 2009; Jarosz et al., 2009; Silverman et al., 2010; Ishijima et al., 2012). In the present study we grew *C. albicans/S. gordonii* polymicrobial biofilms utilizing a synthetic saliva medium [basal medium mucin (BMM)] to more closely mimic physiological conditions found by these microorganisms within the oral cavity. Our goal was to study the morphological and architectural characteristics, as well as the antifungal susceptibility profiles associated with mixed oral *C. albicans/S. gordonii* biofilms grown in this artificial saliva medium as compared to those formed using traditional microbiological media.

## Materials and Methods

### Composition and Preparation of BMM Synthetic Saliva Medium

Preparation of BMM synthetic saliva followed the protocol from Wong and Sissons (Wong and Sissons, 2001), and consists of the following: 2.5 g partially purified pig gastric mucin, 5 g protease peptone (PP), 5 g yeast extract (YE), 33.5 mmol KCl, 2.5 mg haemin, 1 mg menadione, 1 mmol urea, and 1 mmol arginine diluted in a liter of Millipore water and sterilized in an autoclave.

### Strains and Growth Conditions

The strains of microorganisms used were *C. albicans* wild-type strain SC5314 and *S. gordonii* wild-type strain Challis DL1.1. *C. albicans* was regularly cultured on yeast peptone dextrose (YPD) agar plates aerobically at 37°C. *C. albicans* suspension cultures were routinely grown in 20 ml of YPD medium in an orbital shaker (150–180 rpm) at 28°C overnight. Cells were harvested by centrifugation (5000 × *g*, 5 min) the supernatant was removed and the pellet washed with sterile PBS followed by vortexing cells and centrifugation (two times), then resuspended in the desired media for biofilm growth and counted using a hemocytometer. Dilutions were made to obtain a final suspension of 1.0 × 10^6^ cells/ml in the corresponding medium, to be seeded for biofilm formation in the wells of microtiter plates (see below).

*Streptococcus gordonii* was regularly cultured on Tryptone Soy Agar with 5% sheep blood plates, anaerobically inside of a CO_2_ incubator. Suspension cultures of *S. gordonii* were grown in 20 ml of Todd-Hewitt Broth + 0.02% w/v Yeast Extract (THB + 0.02% YE) media, without shaking inside a 5% CO_2_ incubator for 16 h at 37°C. After 16h incubation, 100 μl from the suspension culture were aspirated and inserted into 10 ml of fresh THB + 0.02% YE media and shaken in orbital shaker (150–180 rpm) for 3 h at 37°C. Bacterial cells were then harvested by centrifugation (5000 × *g*, 5 min), the supernatant was removed and the pellet washed with sterile PBS followed by vortexing cells and centrifugation (two times). Bacterial cell concentrations were calculated measuring OD_600_ with a spectrophotometer. Finally, dilutions were performed in order to obtain a final concentration of 1.0 × 10^7^ cells/ml in desired media for growth of biofilms (see below).

### Drugs

A stock solution of fluconazole (Hospira, Lake Forest, IL, USA) prepared in sodium chloride for injection at 2 mg/ml was obtained and stored at 4°C until used. Amphotericin B was obtained in solution at 250 μg/ml (Gibco Life Technologies, Grand Island, NY, USA) and stored at -20°C until used. Caspofungin (Merck and Co., Inc., Whitehouse Station, NJ, USA) was obtained as a powder and was stored at 4°C; a stock solution was prepared in PBS at 2 mg/ml the same day before its addition to well plates. Clindamycin (RPI, Corp., Prospect, IL, USA) was obtained as a powder; a stock solution was prepared in de-ionized water at 10 mM and stored at 4°C until used.

### Biofilm Formation in 96-Well Microtiter Plates

One hundred μl of the prepared dilutions with single or mixed microorganisms (1 × 10^6^ cells/ml for *C. albicans*, 1 × 10^7^ cells/ml for *S. gordonii)* in RPMI 1640, THB + 0.02% YE, 1:1 v/v RPMI/ THB + 0.02% YE media or BMM Synthetic Saliva were pipetted into each well of a 96-well (flat bottom) microtiter plate (Corning^®^ Incorporated, Corning, NY, USA). The selected cell concentrations were based on previous reports on polymicrobial biofilms (Harriott and Noverr, 2009; Cugini et al., 2010; Diaz et al., 2012; Filkins et al., 2015). The plates were then incubated for 24 h inside a 5% CO_2_ incubator at 37°C. After incubation, the supernatant was removed and samples were washed twice with 100 μl PBS. PBS was aspirated and the viability of cells within the biofilms was estimated by adding 100 μl of 1:10 v/v Presto Blue^TM^ Cell Viability Reagent (Invitrogen^TM^, Carlsbad, CA, USA) in 1:1 v/v RPMI/ THB + 0.02% YE media and incubated for 30 min inside a 5% CO_2_ incubator at 37°C. Finally, 80 μl from each well were transferred into a new 96-well plate for fluorescent readings. The microtiter plate reader (BioTek^®^ Synergy^TM^ HT, Winoosky, VT, USA) was set to measure fluorescence at 530/25 nm excitation and 590/35 emission.

### Kinetic Studies on the Formation of Mixed Biofilms on 96-Well Microtiter Plates

After seeding fungal and bacterial cells on 96-well microtiter plates (as described above), samples were collected every 4 h for a period of 24 h, in triplicates. Plates were washed twice with PBS. Cell viability was measured by adding 100 μl of 1:10 v/v Presto Blue^TM^ as described above.

### Bright-Field Microscopy

One hundred μl of crystal violet solution (0.6 g crystal violet prepared in 10 ml isopropanol, 10 ml methanol, 180 ml Millipore water) were added to wells containing the biofilms and removed after 1 min. Excess stain was removed by washing biofilms once with PBS. Samples were directly observed on the 96-well plate using a 40x objective in an inverted system microscope (Westover Scientific, Mill Creek, WA, USA) equipped for photography. The images were processed for display using Micron software (Westover Scientific).

### Scanning Electron Microscopy

For scanning electron microscopy (SEM), biofilms were grown in 6-well plates (Corning^®^) with 6 ml of *C. albicans* at 1 × 10^6^ cell/ml, *S. gordonii* at 1 × 10^7^ cell/ml or mixed at the same final cellular concentrations and incubated in a 5% CO_2_ incubator for 24 h at 37°C. Biofilms were then fixed with a solution of glutaraldehyde (2.5% [wt/vol])-0.1 M sodium calcodylate buffer at pH 7.4 for 2 h at 37°C. Following fixation, the samples were treated with osmium tetroxide solution (1% [wt/vol])-0.1 M sodium calcodylate buffer at pH 7.4 for 2 h at room temperature. Samples were then rinsed with water and washed in a graded series of ethanol solutions (a step gradient of 30, 50, 70, and 90% in water for 10 min per step) ending with 100% ethanol. Before visualization, samples were dried overnight in a vacuum dryer and subsequently coated with a 60:40 gold-palladium alloy, with an approximate thickness of 883 Å using a sputter coater. Samples were observed using a JEOL JSM-6610 Scanning Electron Microscope (JEOL USA, Inc., Peabody, MA, USA). The images were processed for display using Photoshop software (Adobe, Mountain View, CA, USA).

### Confocal Scanning Laser Microscopy

Biofilms were grown using 6-well plates (same as for SEM) and incubated in a 5% CO_2_ incubator for 24 h at 37°C. For confocal scanning laser microscopy (CSLM), biofilms were stained in the dark in the following order: at 37°C for 30 min with 25 μg/ml concavalin A-Alexa Fluor^®^ 488 conjugate (Molecular Probes, Eugene, OR, USA), at room temperature for 30 min with 1X FilmTracer^TM^ SYPRO^®^ Ruby Biofilm Matrix Stain (Molecular Probes), and for 10 min at 37°C with 300 nM 4′,6-diamidino-2-phenylindole, dihydrochloride (DAPI, Molecular Probes). After incubation the supernatant was removed and the biofilms rinsed with 2 ml of PBS to remove non-adhered cells. Lastly, biofilms were gently washed with PBS for removal of excess stain. Samples were viewed using a LSM 510 upright confocal microscope (Carl Zeiss, Thornwood, NY, USA) with an Achroplan 63x-oil objective, using excitation/emission wavelengths of 358/461 nm for blue fluorescence, 495/519 nm for green fluorescence and 450/610 nm for red fluorescence. Pictures were analyzed using AutoQuant X2 (Media Cybernetics, Rockville, MD, USA); additionally 3-D images were made using IMARIS 6.4 software (Bitplane, St. Paul, MN, USA). The images were processed for display using Photoshop software (Adobe, Mountain View, CA, USA). Production of exopolymeric matrix material was estimated using image processing software Fiji by measuring intensity of the red color in the confocal images (Schindelin et al., 2012).

### *In Vitro* Biofilm Antifungal Susceptibility Testing

For biofilm inhibition assays, cell cultures were processed as mentioned previously but the final concentrations for microorganisms were prepared at *C. albicans* at 3 × 10^6^ cell/ml and for *S. gordonii* at 3 × 10^7^ cell/ml for *S. gordonii* in 1:1 v/v RPMI/ THB + 0.02% YE media and BMM Synthetic Saliva. Then 33.3 μl of the prepared cell dilutions of single and mixed microorganisms were pipetted into each well of a 96-well microtiter plate. For monotherapy assays, 33.3 μl of media of choice (1:1 v/v RPMI/ THB + 0.02% YE media or BMM Synthetic Saliva), finally, 33.3 μl of drug solutions at different concentrations were pipetted into the desired wells. Positive controls were no drug control (only medium of choice and cells), and negative controls were wells where cells within the biofilms had been killed with 33.3 μl of 10% Triton^®^ X-100 (Fisher Bioreagents^®^, Fair Lawn, NJ, USA). For combination therapy (antifungals plus clindamycin), 33.3 μl of the prepared cell dilutions of single and mixed microorganisms were pipetted into each well, plus 33.3 μl of antifungal (diluted in either 1:1 v/v RPMI/ THB + 0.02% YE media or BMM Synthetic Saliva), along with 33.3 μl of Clindamycin solution (diluted in either 1:1 v/v RPMI/THB + 0.02% YE media or BMM Synthetic Saliva) at desired concentrations. Final concentrations tested for each drug were: Fluconazole at 0.5, 0.25, 0.125, 0.0625, and 0.03125 mg/ml; Amphotericin B at 16, 4, 1, 0.25, and 0.06 μg/ml; Caspofungin at 16, 8, 4, 2, and 1 μg/ml; and Clindamycin at 100, 10, 1, 0.1, and 0.01 μM. After incubation for 24 h, microtiter plates were washed and processed using the Presto Blue^TM^ assay as described above. From these results, sessile minimum inhibitory concentration (SMIC) values for each drug were determined at both 50 and 80% inhibition.

Antimicrobial susceptibility testing was also performed by adding drugs at desired concentrations to preformed single and mixed species biofilms grown in different media (1:1 v/v RPMI/ THB + 0.02% YE media or BMM Synthetic Saliva), which were then incubated for an additional 24 h in the presence of drugs. Briefly, wells of microtiter plates were seeded with microorganisms and incubated for 24 h to allow for biofilm formation. Drugs were diluted in either 1:1 v/v RPMI/ THB + 0.02% YE media or BMM Synthetic Saliva and added to the preformed biofilms (after washings) at the following final concentrations: Fluconazole at 1, 0.5, 0.25, 0.125, 0.0625 mg/ml; Amphotericin at B 16, 4, 1, 0.25, 0.0625 μg/ml; Caspofungin at 16, 8, 4, 2, 1 μg/ml and Clindamycin at 100, 10, 1, 0.1, 0.01 μM. After incubation for an additional 24 h, microtiter plates were washed and processed using the Presto Blue^TM^ assay as described above. Additionally, SMIC values for each drug were determined at both 50% and 80% inhibition against preformed biofilms.

### Statistics

Viability assays of single and dual-species biofilms were performed with 11 replicates for each growth condition assessed. The data was analyzed using Prism (GraphPad, La Jolla, CA, USA) and the differences were considered statistically significant if *P* < 0.05 by one-way ANOVA test. Dunnett’s multiple comparison was performed considering RPMI 1640 as the medium of reference for *C. albicans* biofilms; THB + 0.02% YE for *S. gordonii* biofilms; and 1:1 mixture of RPMI 1640 and THB + 0.02% YE for mixed biofilms; for comparison against all other growth media, and differences were considered statistically significant if *P* < 0.05. Biofilm kinetic studies were performed in triplicate for each sample type at every time point. Drug susceptibility assays were performed in triplicate, data was normalized with respect to the average of 6 positive control samples (no drug sample) considered as 100%, and the average of 6 negative control samples (treated with 10% Triton X-100), considered as 0%. For each sample the average of 3 replicates was subtracted by the average of the positive controls, and divided by the difference of positive and negative controls, finally multiplied by a 100 ((Sample average- POS Average)/(POS Average-NEG Average))^∗^100.

## Results

### Growth Characteristics of *C. albicans* and *S. gordonii* Single and Dual Species Biofilms in Synthetic Saliva as Compared with Traditional Microbiological Media

We used a 96-well microtiter plate model for biofilm formation to study single and mixed-species biofilm development in BMM synthetic saliva as compared to traditional laboratory media. For comparison purposes, RPMI 1640 media was chosen for biofilm growth as it is widely used for the formation of *C. albicans* biofilms, and similarly, THB + 0.02% YE media is conventionally used for growth of *Streptococci* biofilms (Loo et al., 2000; Pierce et al., 2008). In order to give a fair advantage to both microorganisms to grow within a dual species biofilm, we also tested a mixture of RPMI 1640 and THB + 0.02% YE at a 1:1 ratio. Metabolic activity of the cells within the resulting biofilms was measured using Presto Blue^TM^, which is able to measure viability of both bacterial and fungal microorganisms (Deepe and Buesing, 2012; Potapova et al., 2013). The Presto Blue^TM^ reagent was used in a 1:10 dilution of 1:1 media, to keep consistency between both cell types, regardless of growth as a mono- or dual- species biofilm.

**FIGURE 1:**
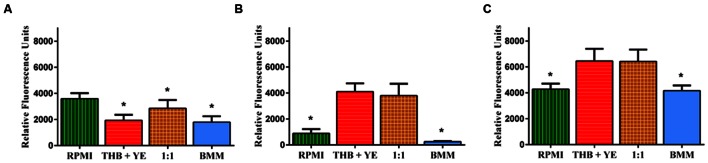
**Extent of biofilm formation in different media as assessed by metabolic activity after 24 h incubation for single species *Candida albicans* (A), *Streptococcus gordonii* (B), and mixed-species (C) biofilms.** Biofilms were grown in RPMI 1640, THB + 0.02% YE, 1:1 v/v RPMI/ THB + YE or BMM synthetic saliva (BMM) in 96-well microtiter plates for 24 h. Viability was measured by Presto Blue^®^ fluorescence Error bars represent standard deviations. Data was analyzed by Dunnett’s multiple comparison test, considering RPMI 1640 as the medium of reference for *C. albicans* biofilms; THB + YE for *S. gordonii* biofilms; and 1:1 mixture of RPMI 1640 and THB + 0.02% YE for mixed biofilms. ^∗^ represents statistically significant differences as compared to the medium of reference *P* < 0.05.

As expected in the case of monospecies biofilms, the use of microorganism-specific media (RPMI for *C. albicans* and THB + 0.02% YE for *S. gordonii*) resulted in the production of highly proliferative mono-species biofilms with increased readouts for metabolic activity for the corresponding microorganism. (**Figures 1A,B**). Similarly, of all media tested, a 1:1 mixture of RPMI 1640 and THB + 0.02% YE as well as THB + 0.02% YE resulted in the most robust generation of mixed fungal/bacterial biofilms (**Figure 1C**). This was also expected, as this media combination should support the growth of both cell types. These results permitted us to consider RPMI 1640 as the medium of reference for *C. albicans* biofilms, THB + 0.02% YE as the medium of reference for *S. gordonii* biofilms and the 1:1 mixture of RPMI 1640 and THB + 0.02% YE as the medium of reference for mixed biofilms for further statistical analyses (**Figures 1A–C**). The use of non-species specific media (i.e., bacterial media for *C. albicans* and fungal media for *S. gordonii*) resulted in less robust biofilms (**Figures 1A,B**). *P* < 0.05 Dunnett’s test. The extent of biofilm formation by *C. albicans* grown in 1:1 mixture of RPMI 1640 and THB + 0.02% YE resulted in intermediate values compared to those obtained using either RPMI 1640 or THB + 0.02% YE media alone (**Figure 1A**). *P* < 0.05 Dunnett’s test. *S. gordonii* biofilms on the other hand, had similar growth in both specific medium THB + 0.02% YE and 1:1 mixture of RPMI 1640 and THB + 0.02% YE, while they grew poorly in both RPMI 1640 and BMM synthetic saliva (**Figure 1B**). *P* ≥ 0.05 Dunnett’s test. In mixed biofilms, higher metabolic readings when directly compared to single species biofilms grown in the same type of media (**Figures 1A–C**) seem to point to synergistic interactions between bacterial and fungal cells. As expected, 1:1 media mixture yielded the most robust (highest metabolic activity) dual-species biofilms. However, interestingly when mixed species biofilms were grown in THB + 0.02% YE, in theory favoring *S. gordonii* growth, results indicated comparable viability readings to those grown in 1:1 media (**Figure 1C**). *P* ≥ 0.05 Dunnett’s test. This suggests that *S. gordonii* is capable of supporting *C. albicans* growth in a not so favorable environment for the fungus when grown together with the bacterium, also pointing to synergistic interactions. As seen in **Figure 1**, for both single- and mixed-species biofilms formed using BMM synthetic saliva, readings of metabolic activity were generally lower compared to similar biofilms formed using traditional microbiological media, and this was particularly noticeable in the case of *S. gordonii* single species biofilms. However, we note that mixed biofilms formed in synthetic saliva displayed metabolic activity similar to those formed in RPMI medium, and demonstrated a synergistic effect as compared to their mono-species counterparts (compare **Figures 1C** to **1A,B**). Overall, these data indicates that BMM synthetic saliva supports biofilm formation by the two microorganisms, although to a lesser extent than traditional microbiological media. Also, results are indicative of synergistic interactions between fungal and bacterial cells leading to the formation of more robust mixed-species biofilms.

**FIGURE 2:**
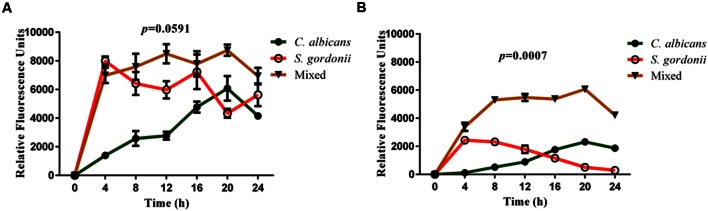
**Kinetic study of single and mixed species biofilms of *C. albicans* and *S. gordonii* grown in 1:1 v/v RPMI/ THB + 0.02% YE media (A) and BMM synthetic saliva (BMM; B).** Cell viability was estimated at 4 h intervals for 24 h using Presto Blue^TM^ fluorescence. Each time point was estimated in triplicates. Error bars represent standard deviations. *P*-values obtained using One-way ANOVA analysis of variance between the three groups (each biofilm type taken as a group). Statistically significant difference between groups is represented by *P* < 0.05.

In a second set of experiments, we performed a series of biofilm kinetic studies to examine formation of both single and mixed species biofilms over a period of time, using both 1:1 media and BMM synthetic saliva. As shown in **Figures 2A,B**, the metabolic activity of *C. albicans* biofilms progressively increases during formation, reaching its peak at 20 h with a slight reduction observed at 24 h in both media and synthetic saliva, thereby indicating maturation. On the other hand, the metabolic activity of *S. gordonii* single-species biofilms is higher at earlier time points (4–12 h), which corresponds with a highly proliferative phase of biofilm development, and gradually reduces at later time points under both growing conditions (**Figures 2A,B**). Also as shown in **Figure 2**, in both 1:1 media mixture (**Figure 2A**), and BMM synthetic saliva (**Figure 2B**) the fluorometric readings for dual-species biofilms are considerably higher than those for single species biofilms at most time points. Of note, in the case of synthetic saliva the metabolic activity of mixed fungal-bacterial biofilms was higher than the sum of each organism individually, particularly during intermediate and late stages of biofilm development, clearly pointing to a synergistic effect. These results were analyzed using One-way ANOVA analysis which demonstrates that single and mixed species kinetics are statistically different, highlighting synergism in mixed species biofilms. Microscopic observations indicated that both single species biofilms colonized the bottom of the wells at a similar rate and demonstrate comparable confluency in both media conditions (Supplementary Figure S1). Bright-field microscopy also allowed us to observe a somewhat distinct shape of *C. albicans* filaments when biofilms were grown in BMM synthetic saliva, which appeared more curvy-like filaments with flaccid appearance (hairy-like) as compared to those formed in media displaying more typical morphological features (Supplementary Figure S1A). *S. gordonii* was able to develop biofilms under both conditions, forming aggregates of cells across the surface (Supplementary Figure S1B). Consistent with metabolic readings, in mixed biofilms total confluency of cells within the wells was reached at around 12 h of incubation, with high interspecies clustering observed particularly as a result of *S. gordonii* accumulation and binding to *C. albicans* filaments (Supplementary Figure S1C).

### Structural Characteristics of Biofilms Formed in BMM Synthetic Saliva as Compared to Those Formed in Traditional Media

**FIGURE 3:**
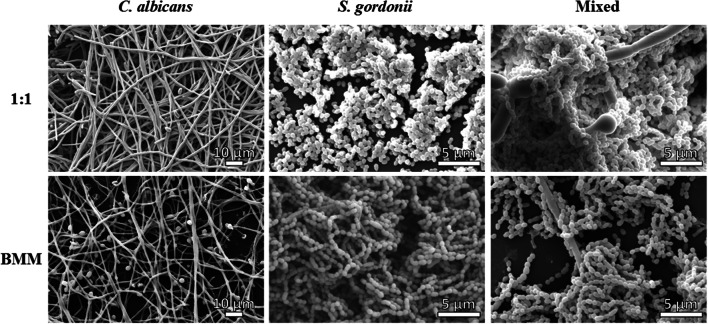
**Scanning electron microscopy (SEM) observations of single species and dual-species *C. albicans/S. gordonii* biofilms grown in 1:1 v/v RPMI/ THB + 0.02% YE media (upper panel) and BMM synthetic saliva (SS, lower panel)**.

**FIGURE 4:**
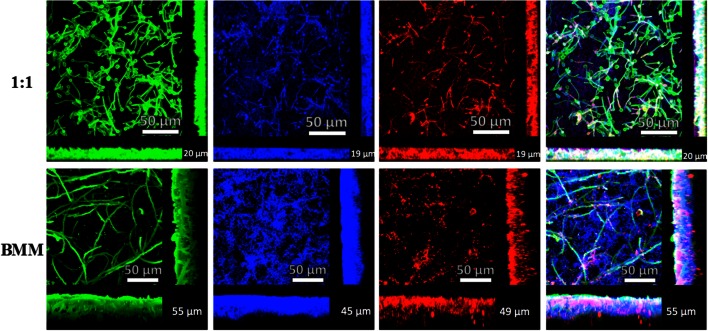
**Characterization of mixed *C. albicans/S. gordonii* biofilms using CSLM and 3D-reconstruction software**. Mixed biofilms grown in 1:1 v/v RPMI/ THB + 0.02% YE media (upper panel) or BMM synthetic saliva (BMM, lower panel). Biofilms were stained with Concavalin A – Alexa Fluor 488 fungal cell wall stain (green), DAPI nucleic acid stain (blue) and FilmTracer^TM^ Sypro^®^ Ruby biofilm matrix stain (red), Numbers at bottom right corner of the picture represent approximate biofilm thickness measured using AutoQuant X2 software.

To analyze the morphological, structural and architectural characteristics and differences between biofilms developed in both media types, we grew biofilms on 6-well plates and visualized them using SEM and CSLM (**Figures 3** and **4**).

Scanning electron microscopy observations revealed that mono-species biofilms formed by *C. albicans* under both media conditions showed predominantly filamentous cells; however, visualization of terminal blastospores and interspersed yeast cells seemed more prevalent in biofilms grown in BMM synthetic saliva, perhaps due to a somewhat lower density of the hyphal mat as compared to biofilms grown in 1:1 medium (**Figure 3**, Supplementary Figures S2A,B). Particularly noticeable was the fact that *S. gordonii* biofilms formed in BMM synthetic saliva showed long streptococcal chains, as compared to densely packed clumps observed in biofilms formed using 1:1 media (**Figure 3** and Supplementary Figures S2C,D). As expected mixed biofilms demonstrated a greater degree of complexity, with the SEM images demonstrating the close interactions between bacterial and fungal cells reflected in high interspecies clustering. In 1:1 media, the biofilms showed highly dense areas of streptococcal cells forming thickly packed aggregates binding to fungal cells. The long chains of streptococcal cells formed in BMM synthetic saliva were found binding predominantly binding to *C. albicans* hyphal elements (**Figure 3**, Supplementary Figures S2E,F). Overall, SEM observations confirmed that, even though significantly lower metabolic activity was detected for biofilms grown in BMM synthetic saliva (**Figures 1A–C**), both fungal and bacterial cells are able to form dense, robust biofilms on the polystyrene surface in synthetic saliva.

Confocal scanning laser microscopy observations confirmed the SEM results and allowed for a more detailed characterization of the main architectural features and three-dimensional characteristics of the mono- and dual-species biofilms under these two conditions (**Figure 4** and Supplementary Figure S3). Mono-species biofilms formed by *C. albicans* were thicker in 1:1 media compared to BMM synthetic saliva, although biofilms formed in the latter medium demonstrated a higher cellular density along with high production of exopolymeric material, seen in red (Supplementary Figure S3A). *S. gordonii* biofilms are much thinner than those formed by *C. albicans*; but they also exhibit increased production of exopolymeric matrix material when grown in BMM synthetic saliva (Supplementary Figure S3B). It is important to mention that a key component of BMM synthetic saliva is mucin, which can bind to Concavalin A, therefore explaining the green fluorescence seen in this sample *S. gordonii* biofilms (Supplementary Figure S3B). Mixed biofilms grown on BMM synthetic saliva are relatively thicker (∼50 μm) compared to biofilms developed on 1:1 media (∼30 μm), and longer hyphae according to the confocal images obtained (**Figure 4**). We note that Presto Blue^®^ fluorometric readings are higher in the case of mixed biofilms grown on 1:1 media, thus potentially indicating lower metabolic activity of sessile cells within the mixed biofilms formed using BMM synthetic saliva despite their increased thickness and density. Furthermore, DAPI and Concanavalin A staining confirmed the previous observations in bright-field microscopy and SEM of streptococcal cells binding to *C. albicans* filaments in mixed biofilms.

### Effect of Antimicrobial Monotherapy and Combination Therapy against Single and Mixed Species Biofilms Formed in BMM Synthetic Saliva and Traditional Microbiological Media

**FIGURE 5:**
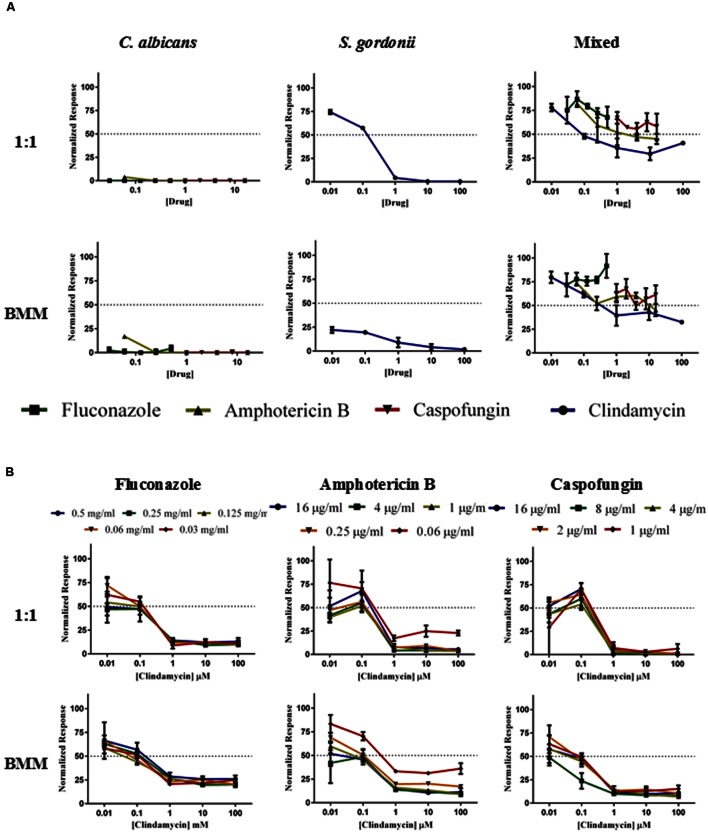
**Inhibition of biofilm formation in single- and dual species *C. albicans/S. gordonii* biofilms formed in 1:1 v/v RPMI/ THB + 0.02% YE media (upper panels A,B) or BMM synthetic saliva (BMM, lower panels A,B) by monotherapy (A) and by antibacterial/antifungal combination therapy (B).** Drugs concentrations were as follows: Clindamycin at 100, 10, 1, 0.1, 0.01 μM; Fluconazole at 0.5, 0.25, 0.125, 0.0625, and 0.03125 mg/ml; Amphotericin B at 16, 4, 1, 0.25, 0.0625 μg/ml, and Caspofungin at 16, 8, 4, 2, 1 μg/ml.

Increased antimicrobial resistance represents one of the hallmarks of the biofilm mode of growth, with important clinical consequences, and the formation of polymicrobial biofilms can complicate the treatment of this type of infections (Jenkinson and Douglas, 2002; Peters et al., 2012). Once the model was fully developed, we performed a series of experiments to examine the efficacy of antimicrobial treatment against the resulting biofilms. These included monotherapy and combination therapy (antifungal plus antibacterial antibiotics) against both single-species and dual species biofilms of *C. albicans/S. gordonii* formed using either synthetic saliva or 1:1 mixed media, and using two different treatment modalities: prevention of biofilm formation and activity against preformed biofilms (**Figure 5**). We used clindamycin and three common antifungal agents. Clindamycin was used, as it is an antibiotic active against gram-positive bacteria and commonly used for the treatment of streptococcal disease, including oral infections (Parks et al., 2015), whereas the antifungals used for these assays were fluconazole, amphotericin B and caspofungin, representatives of each of the major classes of clinically-used antifungals (azoles, polyenes, and echinocandins, respectively; Pfaller, 2012; Pierce et al., 2013). Using the afore-mentioned antifungals in monotherapy for the inhibition of *C. albicans* biofilms proved to be highly effective in 1:1 media (**Figure 5A**, upper panel) and BMM synthetic saliva (**Figure 5A**, lower panel). Likewise, inhibition of *S. gordonii* biofilm formation was effectively achieved at the higher doses of Clindamycin tested in these experiments in both media used (100 and 10 μM; **Figure 5**). As expected, we observed much lower levels of inhibition of mixed *C. albicans/S. gordonii* biofilms using each of the antimicrobials alone (monotherapy modality) irrespective of the growth medium used (**Figure 5A**). Supplementary Table S1 summarizes the resulting SMIC_50_ and SMIC_80_ values for each drug tested in monotherapy experiments against both single- and dual-species biofilm formation.

Using combinatorial treatment, that is adding antifungals plus clindamycin to prevent the formation of mixed biofilms, there was a more effective inhibition of biofilm formation, particularly at the higher doses tested of both drugs, with caspofungin plus clindamycin showing the highest degree of inhibition than any other drug combination (**Figure 5B**). This is particularly noticeable when considering physiological/therapeutic concentrations for each of the antifungals employed in this study, as due to the known recalcitrance of biofilms to azoles, the fluconazole concentrations used are in the mg/ml range and considered to be suprapharmacological. Moreover, although somewhat subtle, for all antifungal/antibacterial combinations we observed a trend of increased resistance for mixed biofilms formed in BMM synthetic saliva as compared to traditional media (**Figure 5B**, lower panel). These results demonstrate that the association of *C. albicans* and *S. gordonii* in dual species biofilms can complicate efforts to prevent biofilm formation, possibly resulting in the development of disease.

**FIGURE 6:**
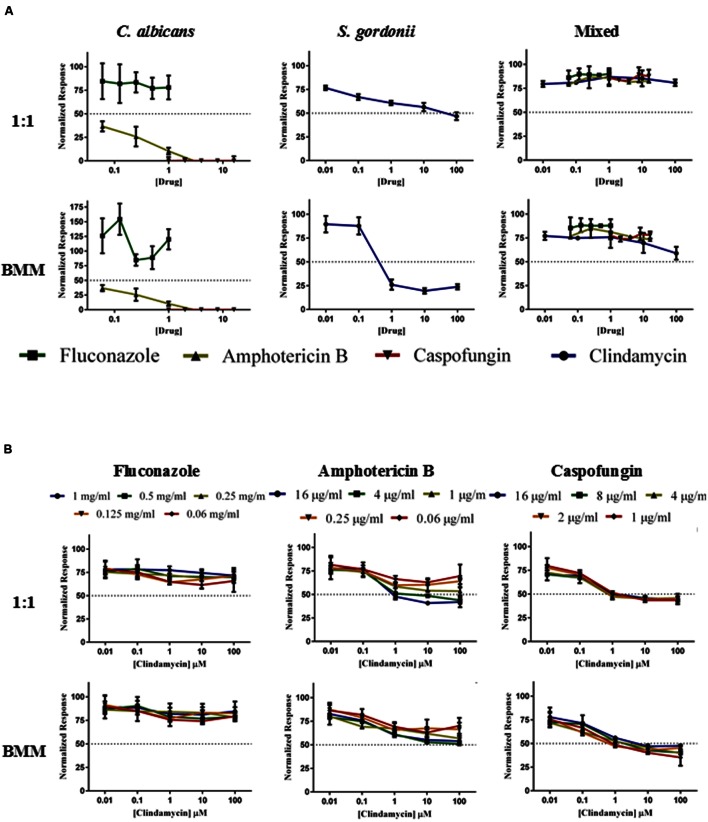
**Antimicrobial susceptibility patterns of preformed single- and dual species *C. albicans/S. gordonii* biofilms formed in 1:1 v/v RPMI/ THB + 0.02% YE media (upper panels A,B) or BMM synthetic saliva (BMM, lower panels A,B) using monotherapy (A) and antibacterial/antifungal combination therapy (B).** Drugs concentrations were as follows: Clindamycin at 100, 10, 1, 0.1, 0.01 μM; Fluconazole at 1, 0.5, 0.25, 0.125, 0.0625 mg/ml; Amphotericin B at 16, 4, 1, 0.25, 0.0625 μg/ml, and Caspofungin at 16, 8, 4, 2, 1 μg/ml.

We also investigated the activity of antibacterial and antifungal antibiotics, either alone or in combination, against single and dual-species preformed biofilms (**Figure 6**). Consistent with previous observations (Pierce et al., 2013), *C. albicans* preformed biofilms displayed increased resistance to antifungal treatment. These fungal biofilms were susceptible to monotherapy with caspofungin at all concentrations tested (16, 8, 4, 2, and 1 μg/ml). Amphotericin B displayed good activity at high doses (16, 4, 1 μg/ml), although we note that these doses are relatively high and generally considered toxic (Pierce et al., 2013). Preformed fungal biofilms show intrinsic resistance to azoles, and as expected fluconazole (even at suprapharmacological concentrations within the mg/ml range) did not have any effect on pre-formed *C. albicans* biofilms as previously reported (Ramage et al., 2001; Pierce et al., 2015; **Figure 6A**). In the case of *S. gordonii*, we observed increased resistance of the preformed bacterial biofilms against monotherapy with clindamycin as compared to the results for inhibition of biofilm formation (compare **Figures 5A** and **6A**); although treatment with clindamycin was more effective against biofilms grown in BMM synthetic saliva (**Figure 6A**). Mixed biofilms, as expected, demonstrated high levels of resistance to all of the drugs when used in monotherapy (**Figure 6A**). Supplementary Table S2 summarizes the resulting SMIC_50_ and SMIC_80_ values for each drug tested in monotherapy experiments against both single- and dual-species preformed biofilms. We then assayed the three previously tested antifungals (fluconazole, amphotericin B, and caspofungin) in combination with clindamycin at various concentrations against preformed dual species biofilms of *C. albicans/S. gordonii* in both 1:1 media and BMM synthetic saliva. As shown in **Figure 6B**, these preformed mixed biofilms are particularly recalcitrant to antimicrobial therapy, irrespective of the media used. The combination of fluconazole and clindamycin proved ineffectual against these bacterial/fungal biofilms. The combinations of clindamycin (at 1, 10, and 100 μM) and either Amphotericin B (at high concentrations), or caspofungin (at lower/physiological concentrations) were able to reduce the metabolic activity in preformed mixed biofilms by 40–50%. Again, these results confirm the higher resistance exhibited by preformed biofilms and most importantly the difficulties in treating established mixed fungal/bacterial biofilms, even when using combinatorial therapy.

## Discussion

Similar to other members of the oral human microbiota, *C. albicans* is able to adapt, live and thrive under the environmental conditions encountered within the oral cavity, where biofilm formation constitutes a major contributing factor to its success as a commensal and opportunistic pathogen (Gendreau and Loewy, 2011; O’Donnell et al., 2015b). Biofilm formation renders fungal cells inaccessible and resistant to antimicrobial treatment and provides protection against the host’s immune system, and as such the impact of complications caused by biofilms on patient care and well-being emphasizes the significance of this study (Pierce et al., 2013, 2015).

*Candida albicans* has the capacity to interact with surrounding bacteria of the human microbiota, occasionally supporting their growth and survival within human tissues (Cramer, 2014; Fox et al., 2014; O’Donnell et al., 2015a), although our current understanding of this phenomenon is limited by the many bacterial species found in the human microbiome that *C. albicans* encounters during and after colonization (O’Donnell et al., 2015a,b). In recent years the ability of *C. albicans* to interact and form mixed species biofilms with members of the oral bacterial microbiota has received increased attention (reviewed in Shirtliff et al., 2009; O’Donnell et al., 2015a). *C. albicans*-bacterial cross-kingdom interactions with important clinical repercussions include those with methicillin-resistant *Staphylococcus aureus* (MRSA) and *Pseudomonas aeruginosa*, which have been shown to communicate with *C. albicans* within a mixed biofilm microenvironment (Hogan et al., 2004; Harriott and Noverr, 2009; Shirtliff et al., 2009; Peleg et al., 2010; Mear et al., 2013; O’Donnell et al., 2015a; Zago et al., 2015). Most recently, (Fox et al., 2014) demonstrated that *C. albicans* is able to produce hypoxic microenvironments within a biofilm to facilitate the growth of anaerobic bacteria such as *Clostridium perfringens*.

It has been reported that under certain circumstances, *C. albicans* virulence can be enhanced by the presence of oral bacteria that may induce the up-regulation of hyphal-specific genes and other virulence-related genes leading to biofilm formation along with the production of secretyl-aspartic proteinases (SAPs), exopolymeric matrix components and adhesins that can contribute to filamentation, colonization and invasive infection (Hawser et al., 1998; Baillie and Douglas, 2000; Diaz et al., 2014; Cavalcanti et al., 2015). In particular, the interactions between *C. albicans* and *S. gordonii*, a gram-positive bacterium that prevails in dental plaque (Marsh et al., 2011), are among the best studied, and the focus of this study. As an early colonizer of the oral mucosa, *S. gordonii* is thought to aid *C. albicans* by providing an adherent surface that further facilitates colonization of oral tissues by *C. albicans*, often resulting in the formation of mixed oral *Candida–Streptococcus* biofilms (Bamford et al., 2009; Diaz et al., 2012; Xu et al., 2014). This interesting relationship suggests intergeneric communication that may involve adhesin-receptor interactions such as bacterial adhesins SspA and SspB with hyphal cell wall receptor Als3p, as well as secretion and/or modulation of quorum sensing molecules, which together lead to synergism for survival as mixed species biofilms (Diaz et al., 2012). For example, Bamford et al. (2009) showed that *S. gordonii* is able to modulate *C. albicans* biofilm formation by preventing proper detection of farnesol by the fungus. This has important implications for pathogenicity, as farnesol is a quorum sensor normally produced by *C. albicans*, which functions as a self-restriction signal to limit biofilm expansion when cells have colonized a surface. Thus, inhibition of farnesol detection leads to production of more robust biofilms, increasing pathogenicity effects and antimicrobial resistance (Hornby et al., 2001; Ramage et al., 2002; Bamford et al., 2009; Lindsay et al., 2012).

Taken into consideration the above-mentioned aspects of oral mixed biofilms, in this study we have developed an *in vitro* model of dual-species *S. gordonii* and *C. albicans* biofilms in 96-well microtiter plate using a physiologically relevant medium of the buccal environment such as BMM synthetic saliva, a medium rich in peptone and yeast extract that was designed to grow oral plaque biofilms (Wong and Sissons, 2001; Sissons et al., 2007), and which closely mimics conditions that microorganisms encounter within the oral microenvironment. We believe this is a good saliva analog as it clearly allows *C. albicans* to filament in our *in vitro* model unlike other synthetic alternatives that do not seem to induce fungal filamentation at 37°C (Arzmi et al., 2015). This is in contrast with previous models that use traditional, nutrient-rich microbiological media, which have been optimized for growth of either bacteria or fungi. We grew single and dual-species (*C. albicans/S. gordonii*) biofilms in 96-well microtiter plates using BMM synthetic saliva medium or traditional microbiological normally used to grow fungal or bacterial biofilms. For estimation of the extent of biofilm formation we used Presto Blue^TM^ which is able to measure metabolic activity of both fungal and bacterial cells (Deepe and Buesing, 2012; Potapova et al., 2013). Our results indicate that BMM synthetic saliva is able to support growth of monospecies biofilms of *C. albicans*, and to a lesser extent *S. gordonii*, although the resulting biofilms are less robust (less metabolically active) than those formed using traditional microbiological media optimized for growth of fungi or bacteria (**Figures 1** and **2**, also Supplementary Figure S1). But perhaps more interesting is the fact that a synergistic effect was clearly manifested in the case of the dual species biofilms formed using the more nutrient-limited synthetic saliva medium, pointing to the existence of beneficial, mutualistic cross-kingdom interactions, as previously reported using different media (Shirtliff et al., 2009). SEM and CSLM provided additional insights into the morphological, structural and architectural characteristics of the resulting biofilms (**Figures 3** and **4**, also Supplementary Figures S2 and S3). The implementation of these advanced microscopy techniques revealed a few notable distinct features of biofilms formed using BMM synthetic saliva as compared to those formed traditional microbiological media. *S. gordonii* biofilms formed in synthetic saliva are composed of long streptococcal chains, as compared to densely packed cellular aggregates observed in biofilms formed using 1:1 media (**Figure 3**). However in the case of mixed species biofilms, and irrespective of morphology, streptococci were found to bind primary to *C. albicans* hyphae within the biofilms, also in agreement with previous reports (Bamford et al., 2009; Silverman et al., 2010; **Figure 3**). CSLM observations corroborated the fact that both media conditions allowed for the formation of mixed biofilms with good cell density. Interestingly, despite lower metabolic readings, mixed biofilms in BMM synthetic saliva display thicker cross-sections and higher biovolume as compared to those formed in traditional microbiological media (**Figure 4**), Moreover CSLM also revealed the higher production of exopolymeric matrix in single species biofilms formed using BMM saliva, although this was not the case in mixed species biofilms.

One of the salient features of biofilms is their increased resistance to antimicrobial treatment, and this problem is further aggravated by the formation of polymicrobial biofilms, with negative clinical repercussions (Haffajee and Socransky, 2001; Lana et al., 2001; Jenkinson and Douglas, 2002; Chukkapalli et al., 2015). Thus, in this study we wanted to examine the resistance properties of biofilms formed in BMM synthetic saliva in comparison to those formed using traditional microbiological media. Our experimental design included the use of monotherapy and combination therapy (antibacterial plus antifungal) against both single- and dual-species biofilms, and two different regimens: one looking at inhibition of biofilm formation and another one at activity against preformed biofilms. Results from these series of experiments confirmed the overall decreased susceptibility of biofilms to antimicrobial treatment, with similar susceptibility patterns observed for biofilms formed using synthetic saliva as opposed to traditional microbiological media. In agreement with previous reports (Jenkinson and Douglas, 2002; Harriott and Noverr, 2009; Peters et al., 2012), results also provided confirmation of the increased resistance of mixed *C. albicans*/*S. gordonii* biofilms as compared to single species biofilms. Thus, for mixed fungal/bacterial biofilms, these results point to the difficulties in preventing biofilm development as well as in treating established biofilms.

Although, more studies are required to unravel the complexity of mixed biofilms, we expect that this model can serve as a platform for further analyses of complex polymicrobial biofilms under growth conditions that more closely resemble those encountered within the oral cavity, and can also be applied to high-content screening of new drug candidates against mixed species biofilms that are urgently needed.

## Author Contributions

DM-J conducted the experiments, wrote the manuscript and analyzed the data. AR contributed to the data analysis. AS and JL-R conceived the study, contributed to data analysis and preparation of the manuscript. All authors read and approved the manuscript for submission.

## Conflict of Interest Statement

The authors declare that the research was conducted in the absence of any commercial or financial relationships that could be construed as a potential conflict of interest.
