# Supplementary material for: An In Vitro Model for Oral Mixed Biofilms of Candida albicans and Streptococcus gordonii in Synthetic Saliva
Source: Front Microbiol. 2016 May 12;7:686. doi: 10.3389/fmicb.2016.00686 (PMC4864667; doi:10.3389/fmicb.2016.00686)
Supplement: Supplementary file 1 [file Data_Sheet_1.PDF]

Supplementary Material

An *in vitro* Model for Oral Mixed Biofilms of *Candida albicans* and *Streptococcus gordonii* in Synthetic Saliva

Daniel Montelongo-Jauregui[1,3], Anand Srinivasan[2,3], Anand K. Ramasubramanian[2,3], and Jose L. Lopez-Ribot[1,3]*

*** Correspondence:** Jose L Lopez-Ribot, Department of Biology, The University of Texas at San Antonio, San Antonio, TX, 78254 USA.

jose.lopezribot@utsa.edu

Supplementary Figures

Supplementary Figure 1.

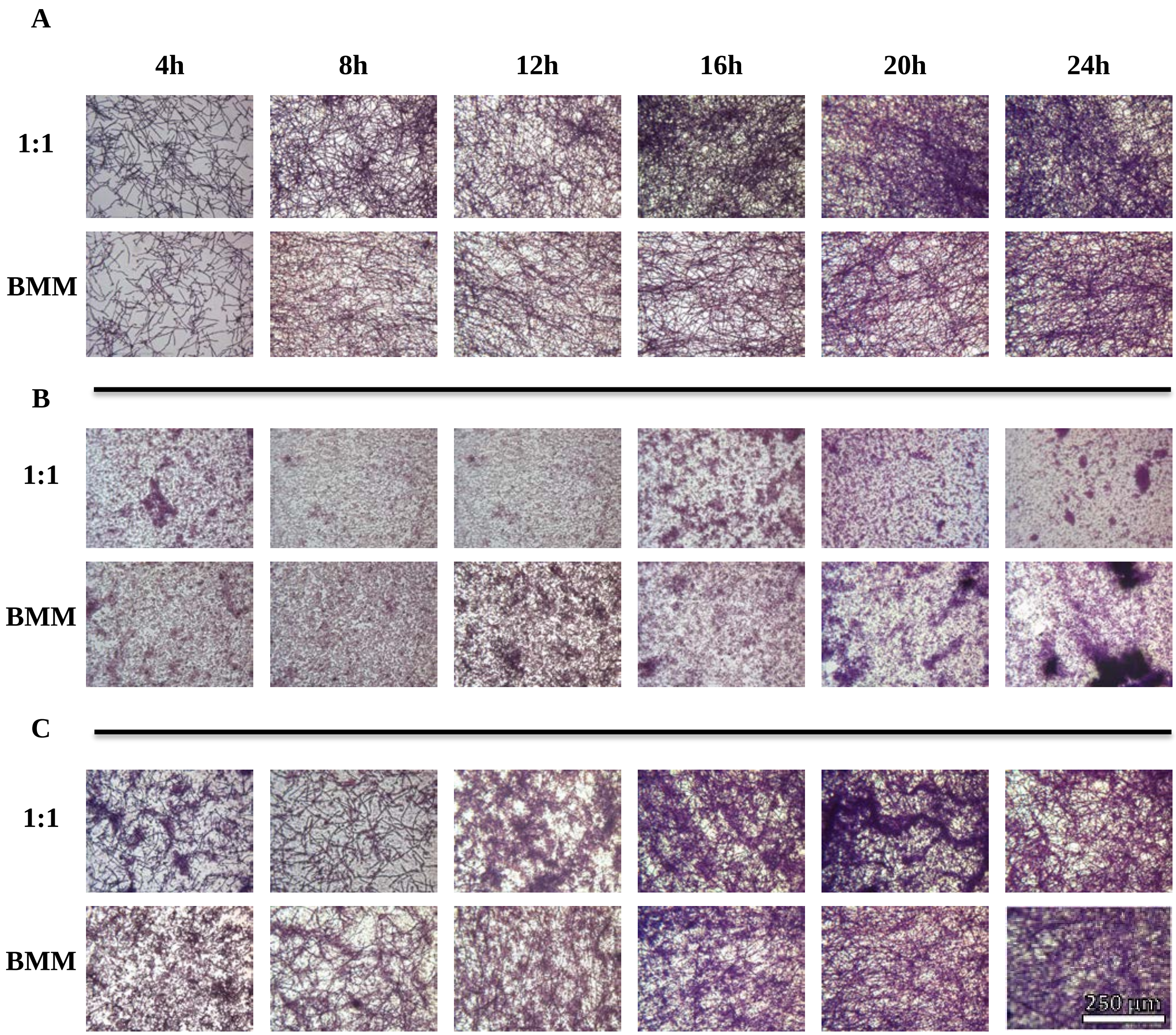

Supplementary Figure 1. Photomicrographs of biofilms formed on the bottom of wells of 96- well microtiter plates during the kinetic study of single- and dual-species biofilms observed under a 40x objective at 4 h intervals using an inverse bright-field microscope. Scale bar applies to all different panels. (**A**) Biofilms formed by *C. albicans* in 1:1 v/v RPMI/ THB + 0.02% YE media; and BMM synthetic saliva; (**B**) Biofilms formed by *S. gordonii* in 1:1 media and BMM synthetic saliva; (**C**) Mixed species biofilms formed in 1:1 media; and BMM synthetic saliva.

Supplementary Figure 2

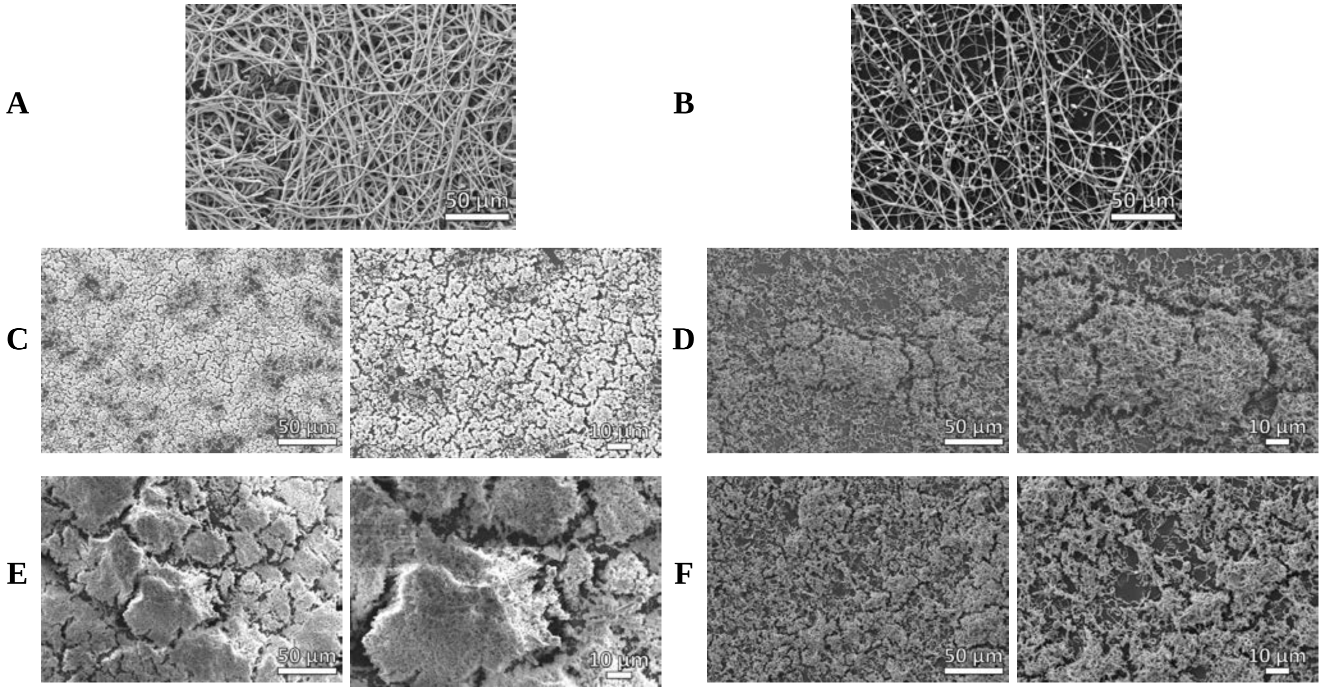

Supplementary Figure 2. SEM images of mature (24 h) single species and mixed species biofilms on a 6-well plate. (**A**) *C. albicans* SC5314 biofilms in 1:1 v/v RPMI/ THB + 0.02% YE media (500x); (**B**) *C. albicans* SC5314 biofilms in BMM synthetic saliva. (500x); (**C**) *S. gordonii* Challis DL1.1 biofilms in 1:1 media. (500x & 1000x); (**D**) *S. gordonii* Challis DL1.1 biofilms in BMM synthetic saliva. (500x & 1000x); (**E**) Mixed *C. albicans* / *S. gordonii* biofilms in 1:1 media. (500x & 1000x); (**F**) Mixed *C. albicans* / *S. gordonii* biofilms in BMM synthetic saliva. (500x & 1000x)

Supplementary Figure 3

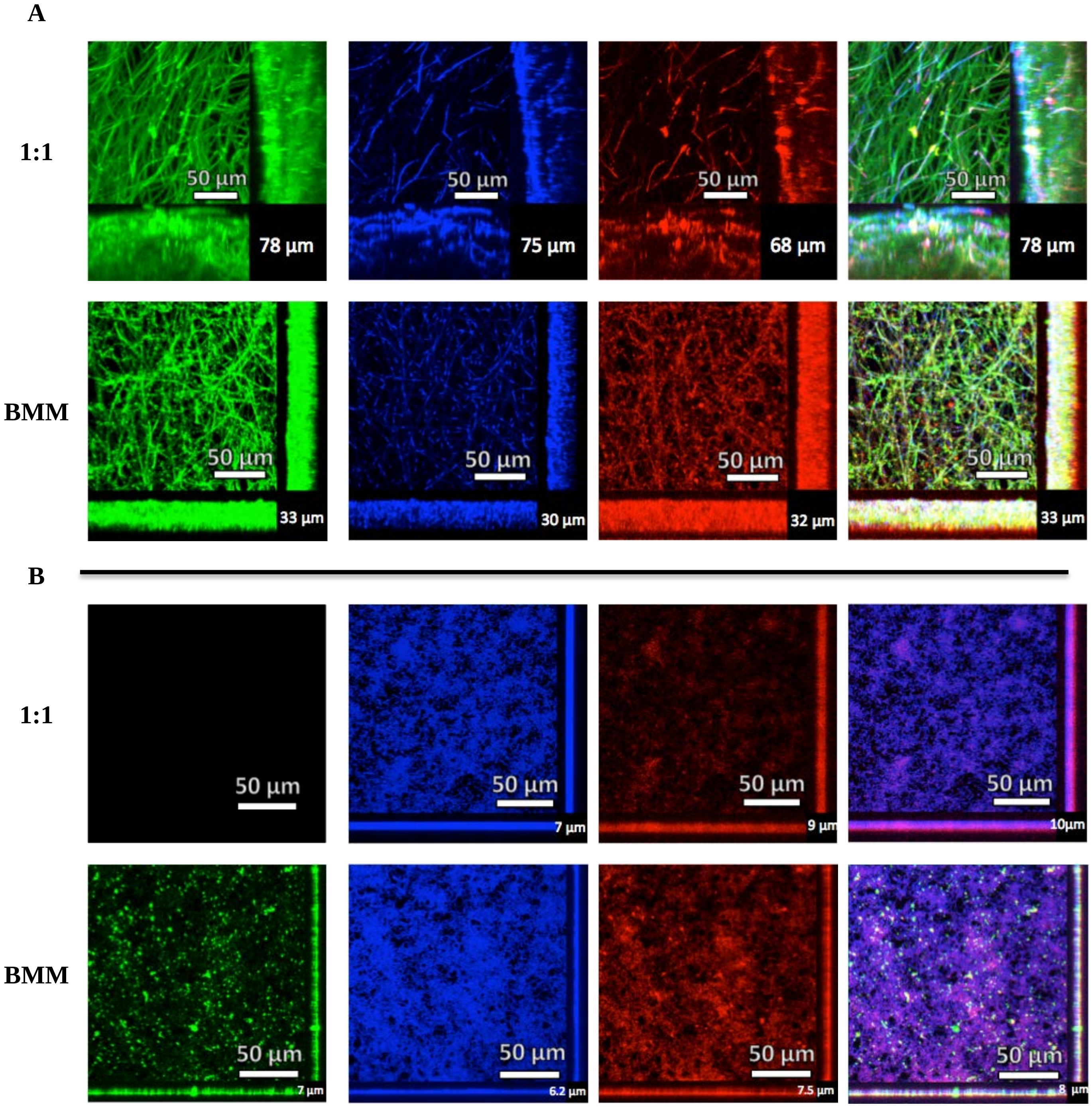

Supplementary Figure 3. Single species biofilms stained using Concavalin A – Alexa Fluor 488 (green), DAPI (blue) and FilmTracer TM Sypro® Ruby biofilm matrix stain (red) and visualized by CSLM. (**A**) *C. albicans* biofilms in 1:1 v/v RPMI/ THB + 0.02% YE media and BMM synthetic saliva; (**B**) *S. gordonii* biofilms in 1:1 media and BMM synthetic saliva. Numbers at bottom right corner of the picture represent approximate thickness measured using AutoQuant software.

2. Supplementary Tables

Supplementary Table 1. $SMIC_{50}$ and $SMIC_{80}$ values of antibiotics used in monotherapy in biofilm inhibition assays against single- and dual-species biofilms, using either 1:1 v/v RPMI/ THB + YE or BMM synthetic saliva.

Biofilm Inhibition	Fluconazole (µg/ml)				Amphotericin B (µg/ml)				Caspofungin (µg/ml)				Clindamycin (µM)			
**Media**	**1:1**		**BMM**		**1:1**		**BMM**		**1:1**		**BMM**		**1:1**		**BMM**	
**Concentration**	SMIC 50	SMIC 80	SMIC 50	SMIC 80	SMIC 50	SMIC 80	SMIC 50	SMIC 80	SMIC 50	SMIC 80	SMIC 50	SMIC 80	SMIC 50	SMIC 80	SMIC 50	SMIC 80
C. albicans	<31.2	<31.2	<31.2	<31.2	<0.06	1	<0.06	1	<1	<1	<1	<1	-	-	-	-
S. gordonii	-	-	-	-	-	-	-	-	-	-	-	-	1	1	<0.01	0.1
**Mixed**	>1000	>1000	>1000	>1000	>16	>16	>16	>16	>16	>16	>16	>16	>100	>100	>100	>100

Supplementary Table 2. $SMIC_{50}$ and $SMIC_{80}$ values of antibiotics used in monotherapy against preformed single- and dual-species biofilms, using either 1:1 v/v RPMI/ THB + YE or BMM synthetic saliva.

Preformed Biofilms	Fluconazole (µg/ml)				Amphotericin B (µg/ml)				Caspofungin (µg/ml)				Clindamycin (µM)			
**Media**	**1:1**		**BMM**		**1:1**		**BMM**		**1:1**		**BMM**		**1:1**		**BMM**	
**Concentration**	SMIC 50	SMIC 80	SMIC 50	SMIC 80	SMIC 50	SMIC 80	SMIC 50	SMIC 80	SMIC 50	SMIC 80	SMIC 50	SMIC 80	SMIC 50	SMIC 80	SMIC 50	SMIC 80
C. albicans	>1000	>1000	>1000	>1000	<0.06	1	<0.06	1	<1	<1	<1	<1	-	-	-	-
S. gordonii	-	-	-	-	-	-	-	-	-	-	-	-	100	>100	1	>100
**Mixed**	>1000	>1000	>1000	>1000	>16	>16	>16	>16	>16	>16	>16	>16	>100	>100	>100	>100
